# Validity of smartphone pedometer applications

**DOI:** 10.1186/s13104-015-1705-8

**Published:** 2015-11-30

**Authors:** Krystn Orr, Holly S. Howe, Janine Omran, Kristina A. Smith, Tess M. Palmateer, Alvin E. Ma, Guy Faulkner

**Affiliations:** Faculty of Kinesiology and Physical Education, University of Toronto, Toronto, Canada; School of Kinesiology, University of British Columbia, Vancouver, Canada

**Keywords:** Physical activity, Activity monitoring, Self-monitoring, Walking, Running

## Abstract

**Background:**

Given the widespread use of smartphone pedometer applications and the relatively limited number of published validity tests, this study examined the validity of three popular commercial smartphone pedometer applications (i.e., Accupedo, Moves, and Runtastic Pedometer).

**Participants:**

Convenience samples of males and females were recruited for laboratory tests [*n* = 11; mean: aged 24.18 years (±3.06)] and a free-living test [*n* = 18; mean: aged 28.78 years (±9.52)].

**Methods:**

Five conditions were assessed: (a) 20-step test, (b) 40-step stair climbing, (c) treadmill walking and running at different speeds, (d) driving, and (e) 3-day free-living. The Yamax SW-200 pedometer and observed step counts were used as criterion measures.

**Results:**

Analyses identified an unacceptable error percentage in all of the applications compared to the pedometer.

**Conclusions:**

Given the inaccuracy of these applications, caution is required in their promotion to the public for self-monitoring physical activity and in their use as tools for assessing physical activity in research trials.

**Electronic supplementary material:**

The online version of this article (doi:10.1186/s13104-015-1705-8) contains supplementary material, which is available to authorized users.

## Background

Daily physical activity (PA) is crucial to the maintenance of mental and physical health [1]. However, only 15 % of Canadians [2] and possibly as few as 5 % of British adults [3] are achieving government-recommended levels of physical activity, respectively. Self-monitoring can be an effective strategy for increasing PA [4] and with the advent of smartphone technology, the accessibility of self-monitoring has become easier through pedometer applications. More than half of the British and Canadian populations use smartphones and over 50 % carry their phones throughout the day [5]. Most smartphones now contain accelerometers [6, 7], which has facilitated the development of a suite of fitness applications [8].

Previous research has assessed the built-in accelerometer’s ability to categorize movement in both Android and iPhone models. Researcher developed algorithms are over 91 % accurate at categorizing walking, sitting, standing, and stair climbing, regardless of phone type [9–11]. Researchers have also tested the built-in accelerometer’s ability to detect steps. Three free pedometer applications accurately detected steps only at moderate walking speeds (6 km/h; [3]). Furthermore, these applications recorded erroneous steps during a driving test. A limitation of this study was that evaluations were completed on a single participant and no free-living condition was included [5]. In another study, the StepCounter software on the Nokia N96 was validated for cardiac rehabilitation patients (*R*^*2*^ = 0.91 between application and standard pedometer; [12]).

Given the growing popularity of smartphone use in everyday life and the promising accuracy of mobile phone technology in activity detection, it is pertinent to conduct further validation studies. There is a current deficit in rigorous and comprehensive research on pedometer applications. If these applications are valid measures of physical activity, they could be a cost-effective and convenient substitute for researchers evaluating walking interventions. Furthermore, these applications could be beneficial for the general public in accurately self-monitoring physical activity, which is an important basis for behaviour change [4].

Therefore, the purpose of this study was to assess the validity and consistency of the three most downloaded pedometer applications available for iPhone, Android, and Windows operating systems (identified using a search for free pedometer applications available on all devices on AppCrawler in October 2014): Accupedo [13], Moves [14], and Runtastic Pedometer [15]. The validity and consistency of these applications were tested on a variety of phones and compared to a research-grade pedometer [16]. Previous research indicates that the Yamax SW-200 is the most accurate pedometer available, and it was therefore used as the criterion measure [16–19]. Following similar protocols [20, 21], the applications were assessed in relation to the Yamax SW-200 using the following tests: (a) 20-step test, (b) 40-step stair test, (c) treadmill test, (d) a driving test, and (e) a free-living test.

## Methods

### Participants

A sample of 11 and 18 participants (aged 22–50 years) were recruited for the laboratory and free-living tests, respectively (see Table 1 for a full description). The study was approved by the University of Toronto’s Office of Research Ethics. All participants gave written consent prior to taking part in the experiments.

**Table 1:** Participants’ descriptions (i.e., heights, mass, BMI, and age) are presented as a group and by testing condition

Measure	Mean (Standard deviation)		
	Total	Laboratory test	Free-living test
Height (m)	1.71 (0.06)	1.69 (0.04)	1.72 (0.06)
Weight (kg)	69.03 (13.28)	60.39 (6.81)	71.73 (13.85)
BMI (m/kg^2^)	23.30 (3.78; 18.1–32.7)	21.20 (2.34; 18.1–26.8)	24.14 (3.98; 19.0–32.7)
Age (years)	27.07 (8.31; 22–50)	24.18 (3.06; 22–32)	28.78 (9.52; 22–50)

### Instruments

This study used a variety of cellular phones (e.g., iPhone 4s, iPhone 5, Samsung Galaxy s5, Samsung Note, Samsung S4, LG Nexus, and HTC Desire) and Yamax pedometers (SW-200 Digiwalker).

#### Application mechanisms

To better understand the applications, researchers contacted manufacturers to identify: (a) how each application works, (b) what phones the applications were compatible with, and (c) how each application differed from other available pedometer applications. No information was provided regarding application algorithms or how the application differed from other similar applications.

##### *Accupedo*

Accupedo only uses the phone’s built-in accelerometer in its algorithm. The application is designed to work regardless of whether the phone is placed in an individual’s pocket, waist belt, or bag, and is compatible with all smartphones (Accupedo, personal communication, November 6, 2014). No information was provided regarding the algorithm for determining the number of steps.

##### *Moves*

Moves uses both global positioning system (GPS) and accelerometer data in its algorithm. Moves is designed to work regardless of whether the phone is in a bag/purse, pocket, hand, or armband [14]. Moves did not correspond with the authors.

##### *Runtastic*

The Runtastic application works solely by GPS (Runtastic, personal communication, November 6, 2014). Distance traveled is divided by stride length to calculate step counts.

#### Yamax Digi-Walker SW-200

The Yamax [16] pedometer uses a coil spring mechanism to account for steps. Once enough force (i.e., ≥0.35G) is applied to the coil spring from the up-and-down motion of the hip girdle during gait the lever arm deflects, accounting for one step [22].

### Procedure

#### Laboratory testing

Within a controlled laboratory setting the researchers conducted four tests of pedometer application validity: (a) 20-step test, (b) 40-stair test, (c) treadmill test, and (d) driving test. A convenience sample of young adults [mean: 21.20 years (±2.34)] was used for all laboratory tests. In each laboratory test the mobile phone was held in the participants’ hand and the pedometer was placed over their right hip [20, 21].

##### *The 20*-*step test*

The pedometer and the applications were zeroed and the participant completed a 20-step test at a normal walking pace. Participants counted their own steps during the test, which were used as the criterion measure. Error greater than one step was considered unacceptable (i.e., ±5 %; [21]).

##### *The stair climbing test*

This test was included given a previous study [9] was unable to accurately measure stair climbing using a step-count application. Participants climbed 40 stairs (i.e., 20 stairs ascending and 20 stairs descending) one at a time, without skipping steps or resting with both feet on a single stair. Participants counted their own steps during this exercise, which were used as the criterion measure. Error greater than two steps was considered unacceptable (i.e., ±5 %).

##### *The treadmill test*

All treadmill tests were conducted on a Woodway treadmill (model DESMO-EVO). Each participant completed 1 min of walking at 2-, 3-, 4.5-, and 6 km/h; the participant then completed 1 min of running at 8-, 9-, 10-, and 11 km/h [20]. Between each trial participants came to a full stop by standing on the side of the treadmill and zeroed their mobile applications and pedometer (approximately 2 min rest). The researchers counted the participants’ steps for each trial. In order to validate these counts, each trial was filmed using a camera aimed at the participants’ lower body, and recorded step counts were used as the criterion measure. Camera counts, mobile application counts, and pedometer counts were compared. One-way repeated measures ANOVAs were used to compare all applications and pedometers at each speed. Post-hoc Bonferroni-corrected *t*-tests compared each application to the pedometer. Percent error scores were computed to evaluate variability by dividing the number of erroneous steps by the true step count (i.e., video count). Standard error of the difference between the application’s counts and the observed count were calculated.

##### *The driving test*

The driving test has been used in previous studies validating accelerometer use under conditions of external disturbance where no steps were taken [20, 21]. This provides information about the sensitivity of the device. The participants were seated in a motor vehicle while driving on a paved surface for 0.2 km at both 10 and 20 km/h. Both speeds were included because authors’ use of the applications detected an erroneous prediction of walking/cycling when participants were in fact travelling at a slow speed in a motorized vehicle. Given that slow speeds may be common for individuals driving in city traffic, it is pertinent to test validity in this condition. Recorded step counts were compared between applications and the Yamax pedometer.

#### Free-living tests

Participants (n = 18) were asked to run all three mobile applications on their personal mobile phone for 3 days (minimum of 10 h) during the free-living trial. The same instructions were given for using the Yamax pedometer, and wear time was used to ensure each day of data was valid (i.e., ≥10 h). Participants were provided with a Yamax pedometer, the criterion measure, and given a calendar-type log to record all step counts from the applications and the pedometer for three full days (see Additional file 1 Digital Content). Moves data was recorded the following morning because Moves’ website identifies this data as more accurate. This was a limitation because the data recorded from the Moves application represented a full day, as Moves cannot be paused, while the other pedometer data was only representative of a minimum of 10-h per day. The participants were given verbal instructions on how to properly wear and use the pedometer. Participants were encouraged to use their mobile phone as they normally would to promote ecological validity. A one-way repeated measure ANOVA compared each of the step-count measures. Post-hoc Bonferroni-corrected *t*-tests compared each application to the pedometer. All statistical analyses were conducted with SPSS 22.

## Results

### Laboratory tests

For all laboratory tests, GPS and cellular service was consistently available.

#### 20-step test

**Fig. 1:**
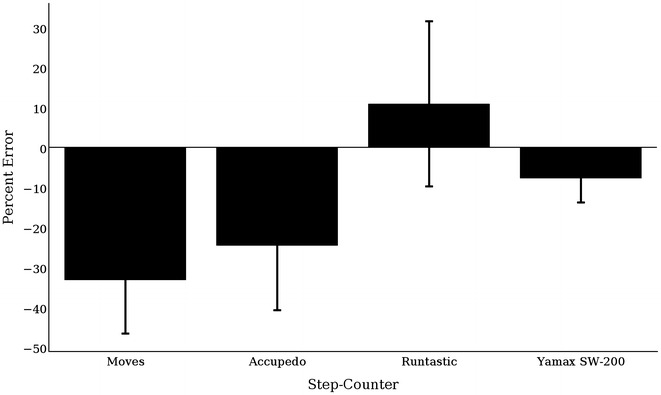
Percent error of each 20-step trial by device compared to the true count (i.e., 20)

The percent error of each trial was computed for each application and the pedometer to assess whether error exceeded ±5 %. All applications had an error greater than ±5 % (Fig. 1). However, the Yamax pedometer showed the smallest error percentage (−0.7 %).

#### 40-step stair climb test

**Fig. 2:**
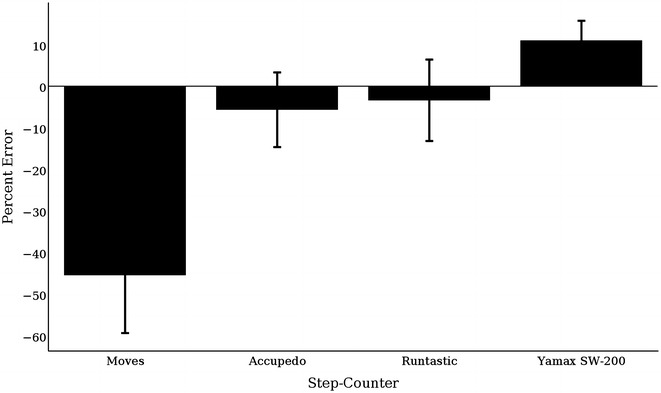
Percent error of each 40-stair climbing trial by device compared to the true count (i.e., 40)

The percent error of each trial was computed for each application and pedometer to assess whether error exceeded ±5 %. Moves, Yamax, and Accupedo had an error greater than ±5 %; Runtastic’s error was −3.41 % (Fig. 2).

#### Treadmill test

Video counts were compared to application and pedometer readings to determine validity. On two occasions, video software failure prevented counts being taken. In these cases, manual counts were used. On two occasions, true count data was not recorded. These participants were excluded from analysis at 2- and 11 km/h. On occasions where applications recorded no steps, the zero point remained in the analyses (see Table 2). On one occasion, the Moves application recorded negative steps (at 9 km/h); this number was rounded to zero steps. On another occasion, Moves recorded 1374 steps in a single bout (at 3 km/h), and this number was replaced with the average Moves count for that trial.

**Table 2:** One-way repeated measures ANOVA results between groups at each treadmill speed are presented

Speed (km/h)	Application													ANOVA
	Moves			Accupedo			Runtastic			Yamax			True count	
	M (SD)	Post-hoc	Zeros recorded	M (SD)	Post-hoc	Zeros recorded	M (SD)	Post-hoc	Zeros recorded	M (SD)	Post-hoc	Zeros recorded	M (SD)	
2	16.30* (33.35)	t(9) = −5.64 *p* < 0.001	7	27.6* (31.41)	t(9) = −4.67 *p* = 0.001	5	30.80* (29.30)	t(9) = −5.50 *p* < 0.001	0	54.40* (24.89)	t(9) = −2.916 *p* < 0.017	0	75.80 (9.53)	F(4,36) = 8.72 *p* < 0.001
3	24.64* (36.11)	t(10) = −6.51 *p* < 0.001	6	21.55* (23.45)	t(10) = −9.61 *p* < 0.001	4	70.64 (42.00)	t(10) = −2.06 *p* = 0.07	1	80.73 (25.88)	t(10) = −1.94 *p* = 0.08	0	94.73 (8.09)	F(4,40) = 14.55 *p* < 0.001
4.5	70.09 (59.79)		4	76.55 (47.60)		2	97.00 (53.93)		0	104.82 (44.27)		1	119.64 (11.41)	F(4,40) = 2.44 *P* = 0.06
6	28.00* (45.55)	t(10) = −8.88 *p* < 0.001	6	103.18 (56.89)	t(10) = −1.90 *p* = 0.09	1	119.00 (55.36)	t(10) = −0.97 *p* = 0.36	0	138.09 (17.33)	t(10) = 0.65 *p* = 0.53	0	134.91 (10.38)	F(4,40) = 18.86 *p* < 0.001
8	80.20 * (74.59)	t(10) = −3.20 *p* = 0.01	3	148.36 (27.26)	t(10) = −0.06 *p* = 0.96	0	143.82 (53.37)	t(10) = −0.32 *p* = 0.75	0	165.91* (14.80)	t(10) = 2.84 *p* = 0.02	0	148.82 (13.92)	F(2.0,19.7) = 7.09 *p* = 0.005
9	80.09* (75.79)	t(10) = −3.94 *p* = 0.003	3	156.36 (28.21)	t(10) = −0.79 *p* = 0.45	0	168.27 (23.39)	t(10) = −0.79 *p* = 0.45	0	170.36* (12.23)	t(10) = 2.39 *p* = 0.04	0	162.55 (15.42)	F(1.4,14.3) = 12.56 *p* = 0.002
10	79.18* (71.88)	t(10) = −4.68 *p* = 0.001	1	167.82 (55.30)	t(10) = 0.24 *p* = 0.81	0	147.82 (57.70)	t(10) = −0.85 *p* = 0.81	0	174.18* (12.58)	t(10) = 2.31 *p* = 0.04	0	163.18 (19.19)	F(4,40) = 7.36 *p* < 0.001
11	130.90 (101.50)		3	166.10 (27.01)		0	144.60 (49.16)		0	175.50 (14.49)		0	162.70 (25.10)	F(1.5,13.5) = 1.218 *p* = 0.32

Post-hoc Bonferroni-corrected *t*-tests were conducted when appropriate (*p* = 0.0125) to the true count (i.e., video recorded)

*p < .05

Data was analyzed for outliers, normality, and sphericity. Runtastic scores at 11 km/h contained one outlier, and were non-normal. The assumption of sphericity was met for the one-way repeated measures ANOVA at all speeds, except 8-, 9-, and 11 km/h. When the assumption was not met, the Greenhouse-Giesser adjusted model was used. When appropriate, Bonferroni-corrected *t*-tests (*p* = 0.0125) compared each application to the observed counts. Results are reported in Table 2.

**Fig. 3:**
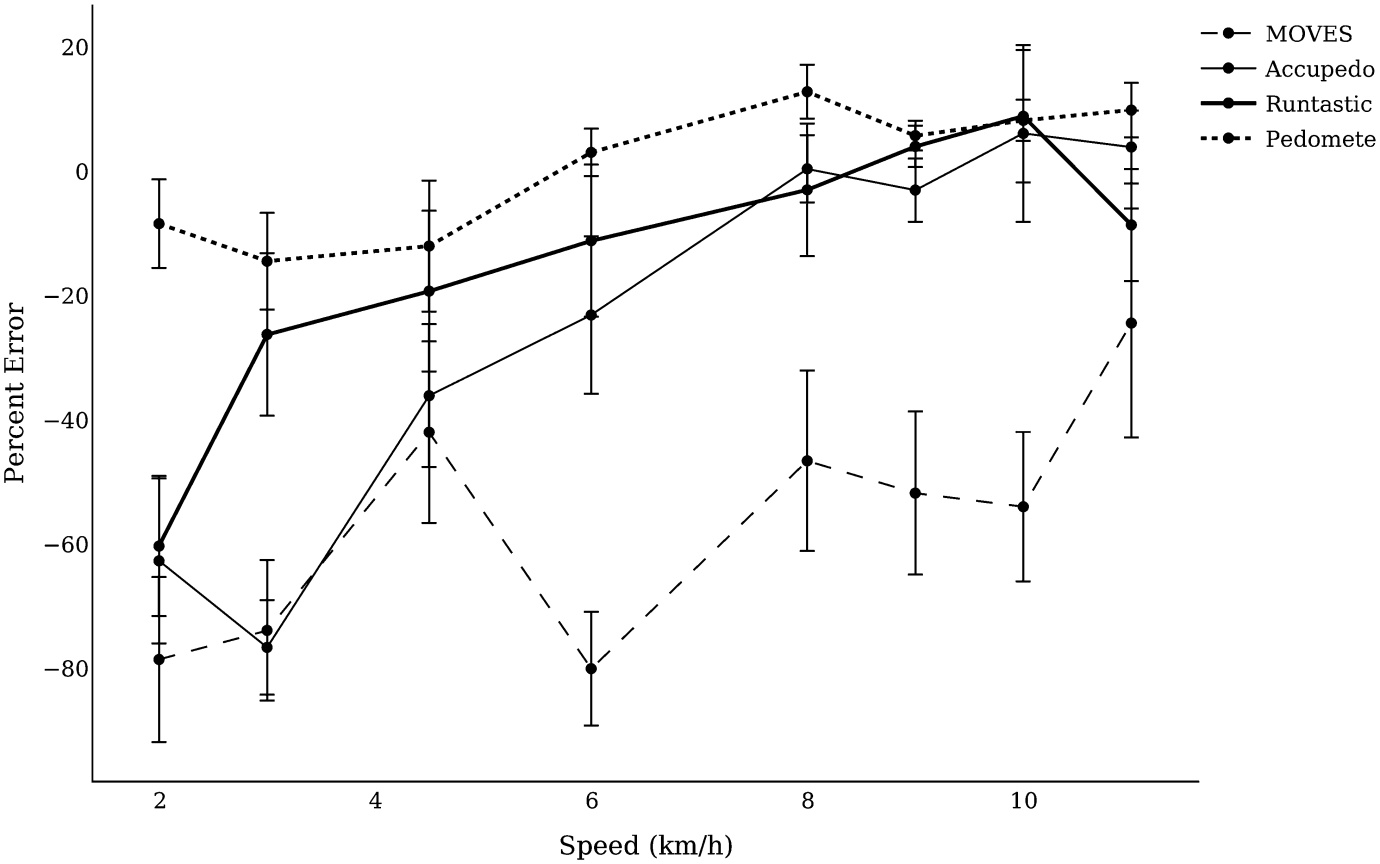
Percent error at each treadmill speed by device compared to the visual count

Percent error scores were also computed to measure the consistency of each application and pedometer. Percent errors were graphed at each speed to illustrate either underestimation (<−10 %), exact (±10 %), or overestimation (>+10 %) of counts compared to the observed (Fig. 3).

To determine the consistency of each application, percent error means and standard deviations were calculated for each application at walking (2–6 km/h) and running speeds (8–11 km/h). Results are reported in Table 3. Higher standard deviations represent lower consistency.

**Table 3:** Pedometer and application reliability are presented as means and standard deviations of the percent error

Application	Test (% error)									
	20-Step (n = 11)		40 Stair (n = 11)		Treadmill walking speed (n = 11)		Treadmill running speed (n = 11)		Free-living (n = 18)	
	M	SD	M	SD	M	SD	M	SD	M	SD
Moves	−29.09	39.55	−45.45	46.26	−68.85	41.75	−45.12	47.32	39.8	29.8
Accupedo	−24.55	53.73	−5.68	8.78	−49.80	41.94	1.33	27.59	21.5	20.4
Runtastic	10.91	68.48	−3.14	9.75	−29.00	43.564	−4.24	29.54	34.5	63.6
Pedometer	−7.23	20.90	11.0	4.34	−8.43	25.33	8.66	12.04		

#### Driving test

Data were analyzed for sphericity and homogeneity of variance. The data violated both assumptions and included outliers. Outliers remained in the data because of small sample size (n = 5). Parametric assumptions were violated; therefore a Friedman test was completed to compare the applications within speeds. There were no significant differences between step-counts recorded by devices at 10 km/h (χ^2^(3) = 6.44, *p* = 0.092) and 20 km/h (χ^2^(3) = 0.636, *p* = 0.88).

#### Free-living tests

Participants recorded an average of 12 h (±0.12 h) of wear time. Data was found to be missing at random and the expectation maximization method was used to impute data for each of the applications, prior to further analysis [23]. A repeated-measures ANOVA found significant differences between the pedometer and applications [F(3,51) = 4.50; *p* = 0.036]. Sphericity was not met; therefore the Greenhouse-Geisser adjusted model was used. Post-hoc Bonferroni corrected *t*-tests revealed each of the applications were significantly different from the pedometer (*p*s < 0.01; see Table 4). Percent error means and standard deviations were calculated for each application compared to the pedometer to determine consistency (see Table 4).

**Table 4:** A one-way repeated-measures ANOVA showed each application to be significantly different from the pedometer

Application	Mean (SD)	Sig. (*p*)
Pedometer	26,466 (±12,795)	–
Accupedo	20,619 (±12,259)	0.000
Moves	19,213 (±12,822)	0.000
Runtastic	23,723 (±16,386)	0.004

Means and standard deviations were derived from all three days of the study. α = 0.05

## Discussion

This is the first study, to the researchers’ knowledge, to examine the quality of three of the most downloaded smartphone pedometer applications: Accupedo, Moves, and Runtastic Pedometer. This study aimed to examine the validity of the applications under various conditions including: controlled driving, walking, running, and free-living in comparison to direct observation and the Yamax SW-200. Overall, the applications were neither valid nor consistent in the sample population under both controlled lab test and free-living conditions.

On the 20-step test none of the applications met the 5 % error threshold and only Runtastic was acceptably accurate on the stair-climbing test. Moderate validity was achieved for Runtastic and Accupedo at higher treadmill speeds (>6 km/h), but did not meet recognized standards [24]. All of the applications performed well during the driving test and were not significantly different from one another.

Under free-living conditions, where intervention studies require the highest validity, the applications were not valid, and commonly underreported steps. Underreporting could have been due to applications being based solely on GPS so they may not have counted steps in areas where there was no service (i.e. subways stations, concrete buildings), poor connectivity, standardized stride length, or drained batteries. Applications that use GPS and accelerometry are thought to be more accurate as they can make use of stride length variability [25]. Poor connectivity and drained batteries also made applications non-functional. Application step counts were not consistently over- or under-estimating pedometer counts. If applications were consistent, users could monitor physical activity and be confident that increases in steps reflected actual improvement despite volume being inaccurate. However, as the applications were highly variable, users cannot accurately monitor increases or decreases in their step counts.

Most published findings suggest that pedometer applications are valid and reliable [e.g., 10, 12, 25], however the current study did not support those results. This can be due to variance in standards, such as phone positioning, application availability, and testing conditions. Additionally, negative results often go unpublished due to publication bias.

### Strengths and limitations

This study had some limitations. Using the Yamax SW-200 as a control measure has some limitations, including over-counting inclined surface and stair steps, stride lengths, and its’ placement may be effected by individual waist circumferences [17]. These limitations could have erroneously inflated our reference step counts. Using step counts from accelerometers in future studies may be beneficial as the mechanisms are more comparable to the smartphone applications (i.e., use built-in smartphone accelerometers). The study used a relatively small convenient sample of adults similar to other pedometer validations [20]. Future research should use larger samples and consider different populations (e.g., older adults or overweight individuals).

The researchers were faced with the issue of controlling the placement of the phone during the tests. It was thought that by holding the phone during laboratory tests, this would be the most gender-neutral and unrestricted location (e.g., differing clothing fits could potentially inhibit movement). Additionally, holding their phones in the laboratory tests and using their phones as normal in the free-living condition increased ecological validity. In a previous study, phones were taped to the low back of participants, which, although accurately counting steps, is not a usual practice [10].

The main strength of this study was its design. The present study included three controlled lab assessment tests (i.e., treadmill, stairs, and step test), in addition to a driving test and free-living condition. Participants in the free-living condition were of varying ages and professions, increasing the ecological validity of this test. Future applications should utilize both GPS and phone accelerometers, however it is suggested that they be developed with the phone developers to improve validity and consistency. This information may be of use to consumers and/or researchers when attempting to identify the most cost-effective tool for physical activity measurement.

## Conclusion

The purpose of this study was to evaluate the quality of smartphone pedometer applications that are freely available for download. Our results suggest that the most commonly downloaded smartphone applications are neither valid nor consistent in measuring step counts. Caution is required in relying on these applications for outcome measures of physical activity within intervention trials. Given the importance of self-monitoring for behaviour change [4], care will also be needed in the promotion of these applications for use by the general public. Further research and development is required in improving the validity of these applications and we anticipate that such improvement will likely come rapidly.

## Additional file

10.1186/s13104-015-1705-8 Instructions and tracking sheet provided to participants in the free-living test.

## Abbreviations

BMI: body mass index
GPS: global positioning system
Km: kilometer
Km/h: kilometers per hour
PA: physical activity
