# Supplementary material for: Validity of smartphone pedometer applications
Source: BMC Res Notes. 2015 Nov 30;8:733. doi: 10.1186/s13104-015-1705-8 (PMC4666074; doi:10.1186/s13104-015-1705-8)
Supplement: Supplementary file 1 — 10.1186/s13104-015-1705-8 Instructions and tracking sheet provided to participants in the free-living test. [file 13104_2015_1705_MOESM1_ESM.docx]

**Tracking Sheet**

**Participant Name: _______________________**

|  | **Wearing my pedometer** | | **Pedometer** | **Accupedo** | **Moves** | **Runtastic** |
| --- | --- | --- | --- | --- | --- | --- |
|  | Time on | Time off |  |  |  |  |
| Tuesday |  |  |  |  |  |  |
| Wednesday |  |  |  |  |  |  |
| Thursday |  |  |  |  |  |  |

Was this an **unusual** working week? If so please give a brief explanation (i.e., sick absent from work, attending a conference)

☐ no ☐ yes: _________________________________________________________

PEDOMETER TASK

1. Familiarize yourself with your pedometer (place on **RIGHT** hip and reset to zero each day)
2. Wear pedometer at least 10 hours/day for 3 consecutive days
3. Download the following Smartphone apps:
   - Accupedo (pedometer)
   - Moves (pedometer)
   - Runtastic (pedometer)
4. Make sure apps are turned on in Settings (turned on for 3 consecutive days):
   - Accupedo (Motion Activity > On, Use Cellular Data > On)
   - Moves (Location > Always, Motion Activity > On, Background App Refresh > On, Use Cellular Data > On)
   - Runtastic (Motion Activity > On, Use Cellular Data > On)
5. Record steps taken at the end of each day (before bed)

*Record Moves daily step count the following morning (record step count text message)

***Take the pedometer off during swimming, showering, and sleeping.***

**HAVE FUN!!**
